# Alterations in prefrontal cortical neuregulin-1 levels in post-pubertal rats with neonatal ventral hippocampal lesions

**DOI:** 10.3389/fnbeh.2022.1008623

**Published:** 2022-12-22

**Authors:** Kenya Watanabe, Osamu Nakagawasai, Syu-ichi Kanno, Satoru Mitazaki, Hiroshi Onogi, Kohei Takahashi, Kei-ichiro Watanabe, Koichi Tan-No, Masaaki Ishikawa, Lalit K. Srivastava, Remi Quirion, Takeshi Tadano

**Affiliations:** ^1^Division of Pharmacology, Faculty of Pharmaceutical Sciences, Tohoku Medical and Pharmaceutical University, Sendai, Japan; ^2^Department of Pharmacy, Fukushima Medical University Hospital, Fukushima, Japan; ^3^Division of Clinical Pharmaceutical Therapy, Faculty of Pharmaceutical Sciences, Tohoku Medical and Pharmaceutical University, Sendai, Japan; ^4^Laboratory of Forensic Toxicology, Faculty of Pharmacy, Takasaki University of Health and Welfare, Takasaki-shi, Japan; ^5^Faculty of Health Science, Tohoku Fukushi University, Sendai, Japan; ^6^Department of Pharmacology, School of Pharmacy, International University of Health and Welfare, Otawara, Tochigi, Japan; ^7^Center for Research on Counseling and Support Services, The University of Tokyo, Bunkyō-ku, Tokyo, Japan; ^8^Douglas Hospital Research Centre, McGill University, Montreal, QC, Canada; ^9^Complementary and Alternative Medicine Clinical Research and Development, Graduate School of Medicine Sciences, Kanazawa University, Kanazawa, Japan

**Keywords:** neonatal ventral hippocampal lesion, neuregulin-1, erbB4, prefrontal cortex, prepulse inhibition, schizophrenia

## Abstract

Genetic studies in humans have implicated the gene encoding neuregulin-1 (NRG-1) as a candidate susceptibility gene for schizophrenia. Furthermore, it has been suggested that NRG-1 is involved in regulating the expression and function of the *N*-methyl-D-aspartate receptor and the GABA_A_ receptor in several brain areas, including the prefrontal cortex (PFC), the hippocampus, and the cerebellum. Neonatal ventral hippocampal lesioned (NVHL) rats have been considered as a putative model for schizophrenia with characteristic post-pubertal alteration in response to stress and neuroleptics. In this study, we examined NRG-1, erb-b2 receptor tyrosine kinase 4 (erbB4), and phospho-erbB4 (p-erbB4) levels in the PFC and the distribution of NRG-1 in the NVHL rats by using immunoblotting and immunohistochemical analyses. Neonatal lesions were induced by bilateral injection of ibotenic acid in the ventral hippocampus of postnatal day 7 Sprague-Dawley (SD)-rats. NVHL rats showed significantly decreased levels of NRG-1 and p-erbB4 in the PFC compared to sham controls at post-pubertal period, while the level of erbB4 did not differ between sham and NVHL rats. Moreover, microinjection of NRG-1 into the mPFC improved NVHL-induced prepulse inhibition deficits. Our study suggests PFC NRG-1 alteration as a potential mechanism in schizophrenia-like behaviors in the NVHL model.

## Highlights

- NVHL rats show significantly decreased NRG-1 and p-erbB4 levels in the PFC.
- Microinjection of NRG-1 into the mPFC improved NVHL-induced PPI deficits.

## Introduction

Excitotoxic neonatal ventral hippocampus lesioned (NVHL) rats have been proposed as a putative animal model for schizophrenia as these animals display post-pubertal neurochemical and behavioral abnormalities analogous to symptoms seen in this neuropsychiatric disorders (Lipska and Weinberger, 2000; Marcotte et al., 2001). For example, post-pubertal NVHL animals are hyper-reactive to stress and amphetamine, display deficits in prepulse inhibition (PPI) of startle and latent inhibition, and impaired social interaction and working memory (Sams-Dodd et al., 1997; Becker et al., 1999; Mitazaki et al., 2020).

Neuregulin-1 (NRG-1) gene and its receptor erb-b2 receptor tyrosine kinase 4 (erbB4) are significant risk factors for schizophrenia (Stefansson et al., 2002; Norton et al., 2006; Silberberg et al., 2006). In the brain, NRG-1 plays multiple roles in synapse formation, activity-dependent synaptic plasticity, and regulation of *N*-methyl-D-asparate, and GABA_A_ receptor subunit expression (Ozaki et al., 1997; Rieff et al., 1999; Liu et al., 2001; Falls, 2003; Hahn et al., 2006; Woo et al., 2007). Postmortem studies have shown that schizophrenia patients have a decreased cortical NRG-1 expression (Bertram et al., 2007; Marballi et al., 2012). Moreover, mutant mice carrying mutations in the NRG-1 or erbB4 gene show schizophrenia-like behaviors such as disruption of PPI, latent inhibition and cognitive deficits (Stefansson et al., 2002; Falls, 2003; Hashimoto et al., 2004; Rimer et al., 2005; O’Tuathaigh et al., 2007). NVHL alters the development and function of the prefrontal cortex (PFC), which receives prominent ventral hippocampus projections, and is implicated in many complex behaviors disrupted by this lesion (Sams-Dodd et al., 1997; Becker et al., 1999; Lipska and Weinberger, 2000; Marcotte et al., 2001; Flores et al., 2005).

Here, we investigated whether PFC neuregulin participates in the behaviors of NVHL rats by assessing the expression of NRG-1 and its receptors, erbB4 and phospho-erbB4 (p-erbB4), in the PFC of NVHL rats using immunoblotting and immunohistochemical. In addition, we assessed the effects of intra-cortical administration of NRG-1 on PPI deficits of NVHL animals.

## Materials and methods

All experiments were performed following the approval of the Ethics Committee of Animal Experiment in Tohoku Medical and Pharmaceutical University (approval numbers: A10016 and A11035) and according to the National Institutes of Health Guide for the Care and Use of Laboratory Animals. Efforts were made to minimize suffering and reduce the number of animals used. Measurements of the behaviors and post-mortem analyses were conducted by an observer blind to treatment conditions. Behavioral testing occurred between 10:00 and 18:00.

### Neonatal ventral hippocampal lesion

Lesions of the ventral hippocampus in pups were performed as previously described (Flores et al., 1996). Pregnant Sprague–Dawley rats at 15 days of gestation were obtained from Japan SLC (Hamamatsu, Shizuoka, Japan), housed individually in 12-h light/dark cycle rooms and fed ad libidum. On post-natal day 7 (PD 7) male pups (15–17 g) within each litter (4–9 males/litter) were randomly divided to sham or lesion status. Pups were anesthetized by hypothermia by placing them on ice for 20 min and were immobilized on a platform fixed on a stereotaxic frame. An incision in the skin overlaying the skull was made and two 1 mm holes were drilled. A needle connected to an infusion pump through a Hamilton syringe was lowered into the ventral hippocampus at the coordinates: AP—3.0 mm ML ± 3.5 relative to bregma and—5.0 relative to the surface of the skull. Ibotenic acid (0.3 μl, 10 μg/μl; Sigma, Chemical Co, St-Louis, MO, USA) in 0.15 M phosphate-buffered saline (PBS) pH 7.4 was infused bilaterally at a flow rate of 0.15 μl/min. Sham operated animals received the same volume of PBS. The needle was withdrawn 2 min after completion of the infusion. Pups were placed under a warming lamp and then returned to their mothers. On PD 21–25, rats were weaned and grouped 2–3 per cage. Experiments were performed on post-pubertal (PD 70–80) animals.

### Drugs

NRG-1 (500 ng or 1000 ng/0.5 μl; R&D Systems, Minneapolis, MN, USA) was dissolved in 0.15 M PBS (pH 7.4), and infused bilaterally in the prelimbic cortex (PrL) (AP + 2.8 mm ML ± 0.5 relative to bregma and—5.0 relative to the surface of the skull) at a flow rate of 0.25 μl/min under anesthesia with pentobarbital Na (50 mg/kg, intraperitoneally administration; Kyoritsu Seiyaku, Tokyo, Japan). The dose for NRG-1 used was calculated based on a previous report (Jalilzad et al., 2019).

### Histology

**FIGURE 1:**
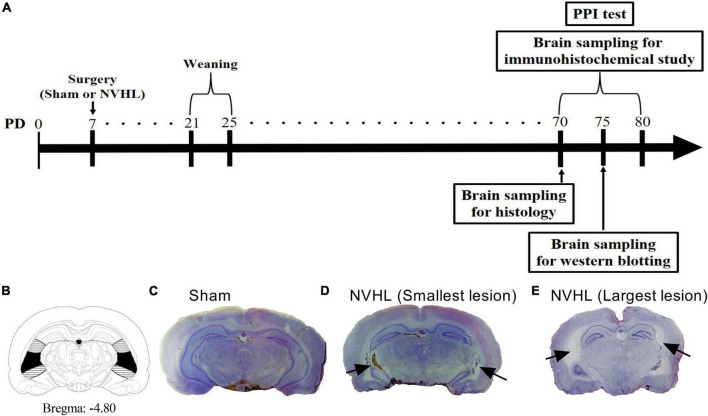
Experimental time course for protocol **(A)**. **(B)** Diagram showing the dorsal to ventral hippocampus. Horizontal bars and blackened areas indicate the largest and smallest lesions, respectively. **(C)** Sham rat. **(D)** NVHL rat (smallest lesion). **(E)** NVHL rat (largest lesion). A typical cresyl violet stained coronal sections at the level of ventral hippocampus of a PD 70 rat indicating ibotenic acid induced neuron loss (arrow) in the ventral hippocampus **(D,E)**.

To assess the location of the lesion, adult rats were sacrificed by decapitation and brains were removed and frozen in 2-methylbutane at –40°C and stored at –80°C until sectioning using a cryostat. We visually confirmed the ventral hippocampal location (Bregma from −4.6 to −6.0 mm) using rat brain stereotaxic coordinates (Paxinos and Franklin, 2013). Coronal sections (Bregma −4.8 mm, 20 μm thick) were mounted onto gelatin-coated slides and stained with cresyl violet. Lesion size was confirmed by digital camera. As shown in Figure 1, bilateral damages including neuronal loss, atrophy and cavitation of the ventral hippocampus was observed in ibotenic acid-treated rats. Animals exhibiting damage in the dorsal hippocampus, thalamus or cortex were excluded from the study. Sham control animals did not show any obvious damage in hippocampal areas.

### Immunohistochemical procedure

The immunohistochemical analysis of NRG-1 was performed on PD 70-80 rats. The rats were anesthetized with Na pentobarbital and perfused through the heart with ice-cold PBS (pH 7.4), immediately followed by a fixative containing 4% paraformaldehyde (Sigma−Aldrich) and 0.2% glutaraldehyde (Nacalai Tesque, Osaka, Japan) in PBS. The brain was then postfixed with the same fixative solution at 4°C for 1 h and then placed in a 20% sucrose-buffered solution at 4°C for 12 h. The tissue was frozen on dry ice and cut into 20-μm-thick coronal sections (Bregma from +4.20 to +2.70 mm) on a cryostat (Leitz, Stuttgart, Germany). The immunohistochemical staining procedure was carried out as previously described (Sutoo et al., 2001; Yabuki et al., 2013). For immunohistochemical staining, sections were incubated with NRG-1 antibody (1:500; Santa Cruz Biotechnology, Santa Cruz, CA, USA, Cat# sc-348) was diluted in PBS containing 1% normal goat serum and 0.1% Triton-X 100 at 4°C for 12 h. This was followed with incubation for 3 h with the secondary antibody FITC-labeled anti-rabbit IgG goat serum (1:200; BioSource International, Inc., Camarillo, CA, USA). The stained sections were mounted in 10% glycerin-PBS, and kept at 4°C in a dark room. The distribution of NRG-1 immunofluorescence intensities was quantitatively analyzed using a modified brain mapping analyzer system (Yamato Scientific Co., Inc., Tokyo, Japan). The background value, such as non-specific fluorescence originating from glutaraldehyde, was subtracted photometrically from the total fluorescence intensity value at each measuring point. Immunohistochemical fluorescence intensities obtained for the regions were indicated relative to that of standard 1mM quinine sulfate. The average NRG-1 fluorescence intensity in each region was obtained from 2 to 3 sections per animal.

### Western blotting

Western immunoblotting analysis was performed using medial PFC samples from rats that had not undergone any behavioral testing. Brain was removed and sectioned on ice, using a rat brain slicer (Muromachi Kikai, Tokyo, Japan), to produce 1 mm thick coronal sections. We visually confirmed the medial PFC location using rat brain stereotaxic coordinates (Paxinos and Franklin, 2013). Protein isolation and western blots were performed as described previously (Takahashi et al., 2018). The blots were incubated with a blocking solution (25 mM Tris-HCl pH 7.4, 137 mM NaCl, 2.68 mM KCl, and 10% skim milk) for 4 h and then with NRG-1 antibody (1:1000; Santa Cruz Biotechnology; Cat# sc-348), erbB4 (1:1000; Santa Cruz Biotechnology; Cat# sc-18), p-erbB4 (1:5000; Santa Cruz Biotechnology; Cat# sc-33040), or β-actin to control for protein loading (1:1000; Cell Signaling Technology; Cat# 4967) overnight at 4°C. The membrane was then washed with blocking solution without skim milk, and horseradish peroxidase-linked secondary antibody (goat anti-rabbit IgG HRP: 1:1000) for 1 h. Protein amount was analyzed by enhanced chemiluminescence with an ECL plus Western blotting detection system (Amersham, Arlington Height, IL, USA). The band density was measured by densitometry (Image-J 1.43μ, National Institute of Health).

### PPI of the acoustic startle response

Prepulse inhibition tests were performed using the SR-LAB system (SR-LAB, San Diego Instruments, San Diego, CA, USA). Rats were placed in the Plexiglas enclosure 24 h after vehicle or NRG-1 administration. Ventilation was provided by a small electric fan that also generated a 70 dB background noise. Measures of both acoustic startle response (ASR) and PPI were obtained in a single session. Rats were allowed to acclimatize to the environment for 5 min before being tested during 42 discrete trials. On the first two trials, the magnitude of the ASR to a 120 dB noise-burst lasting 50 ms was measured. These first two startle noise-bursts were presented in order to habituate the animals to the testing procedure. Therefore, the ASR magnitude of these two trials was omitted from the statistical analysis of the mean ASR amplitude. On the subsequent 40 trials, the startle noise-burst was either presented alone or 100 ms after the presentation of a 30 ms duration prepulse. Prepulse intensity ranged from 3 to 15 dB above background noise and was varied randomly between trials in 3 dB steps. Measures were taken at each of the five prepulse intensities during five trials; animals were randomly presented with the startle noise-burst alone during another ten trials; null trials (background noise alone) were conducted during the other five trials. The same stimulus condition was never presented on more than two consecutive trials. The interval between each trial was programmed to a variable time schedule with an average duration of 15 s (range 5–30 s). A measure of startle response amplitude was derived from the mean of 100 digitized data points collected from stimulus onset at a rate of 1 kHz. PPI was expressed as a percentage based on the mean amplitude of response to the startle pulse-alone trials relative to those recorded under the five prepulse conditions: PPI = 100 − [(mean startle amplitude for prepulse + pulse trials/mean startle amplitude for pulse-alone trials) × 100]%. PPI under the five different prepulse intensities were averaged and used for statistical analysis.

### Statistical analysis

Results are expressed as mean ± standard error of the mean (SEM). The significance of differences was determined by the Student’s *t*-test for two-group comparison, and by a two-way analysis of variance (ANOVA), followed by Tukey–kramer test for the startle amplitude and average PPI data for multigroup comparisons.

## Results

### Histological examination of the ventral hippocampal lesion

Experimental time course for protocol is shown in Figure 1A. As shown in Figures 1B–E, NVHL rats showed bilateral cell loss and cavitation of the ventral hippocampus, while sham-lesioned control animals did not show any obvious damage in ventral hippocampal areas.

### Distribution of NRG-1 fluorescence intensity in the PFC

**FIGURE 2:**
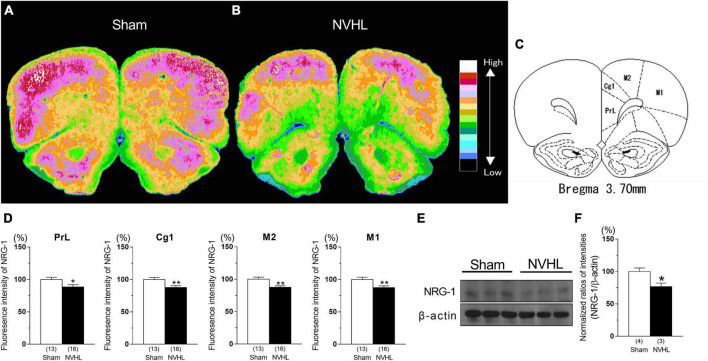
Reduced NRG-1 levels in the PFC in the NVHL rats at post-pubertal period. Immunohistochemical fluorescence intensity for NRG-1 in PFC. **(A)** Sham rat. **(B)** NVHL rat. **(C)** Diagram showing the prelimbic cortex (PrL), cingulated cortex area 1 (Cg1), secondary motor cortex (M2), and primary motor cortex (M1). **(D)** Values for the immunofluorescence intensity for NRG-1 in PrL, Cg1, M2, and M1. The distribution of NRG-1 fluorescence intensity in each rat PFC section was determined by microphotometry and classified into 16 levels [as indicated by color in panel **(A,B)**, with lowest concentration in black and highest concentration in white]. Representative immunoblots probed with antibodies against NRG-1 and β-actin, as indicated **(E)**. Quantification of the normalized values of NRG-1 levels with β-actin **(F)**. Numbers in square brackets indicate the number of animals in each group. Bars represent means ± SEM. *p < 0.05 and **p < 0.01 vs. sham group.

As shown in Figures 2E, F, NVHL rats at post-puberty showed a significant decrease in NRG-1 protein level in the PFC [Student’s *t*-test: *t*(5) = 2.98, *p* < 0.05, Figure 2F]. In addition, as shown in Figures 2A–C, immunohistochemical analyses showed a marked decrease in NRG-1 fluorescence intensity the prelimbic cortex (PrL), cigulated cortex area 1 (Cg1), secondary motor cortex (M2), and primary motor cortex (M1) in the NVHL group compared with the sham group of animals [Student’s *t*-test: PrL: *t*(27) = 2.31, *p* < 0.05, Cg1: *t*(27) = 3.0, *p* < 0.01, M2: *t*(27) = 3.26, *p* < 0.01, M1: *t*(27) = 3.013, *p* < 0.01, Figure 2D].

### Change in phosphorylation of erbB4 level in the PFC of NVHL rats

**FIGURE 3:**
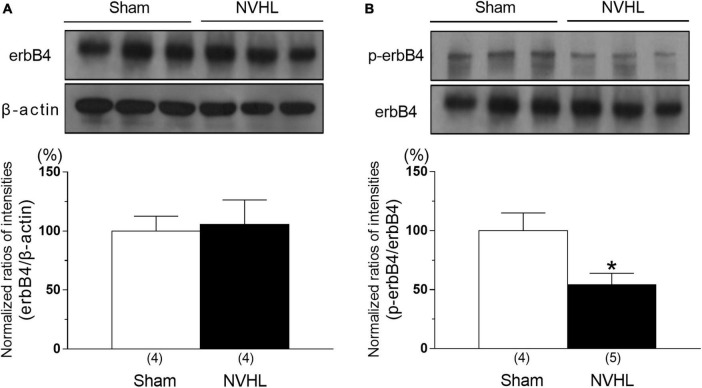
Alternation of erbB4 and p-erbB4 level in the PFC in the NVHL rats at post-pubertal period. Representative immunoblots probed with antibodies against β-actin, erbB4 **(A)**, and p-erbB4 **(B)**, as indicated. Quantification of the normalized values of erbB4 levels with β-actin **(A)** and p-erbB4 levels with erbB4 **(B)**, respectively. Numbers in square brackets indicate the number of animals in each group. Bars represent means ± SEM. *p < 0.05 vs. sham group.

As shown in Figure 3, the immunocontent of phosphorylation of erbB4 level in the PFC of NVHL rats at post-puberty was significantly decreased compared to sham group, while erbB4 level was unchanged between sham and NVHL rats [Student’s *t*-test: *t*(6) = 0.22, *p* = 0.83, Figure 3A; *t*(7) = 2.67, *p* < 0.05, Figure 3B].

### Effects of intra-cortical microinjection of NRG-1 on PPI in post-pubertal NVHL rats

**FIGURE 4:**
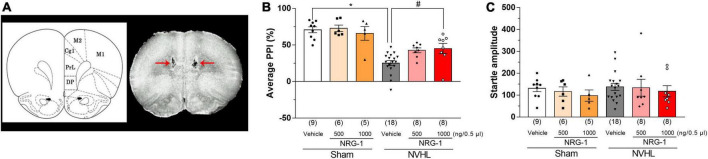
Intra-cortical microinjection of NRG-1 improves NVHL-induced PPI deficits in rats. **(A)** Photograph of cortical microinjection sites of NRG-1. Schematic drawing of coronal brain sections and photograph of a frontal section of the brain in an animal with microinjection are shown in panel **(A)**. Red arrows indicate the microinjection sites of NRG-1 into the mPFC. Effect of NRG-1 on average PPI **(B)** and startle amplitude **(C)** in sham and NVHL rats. Numbers in square brackets indicate the number of animals in each group. Bars represent means ± SEM. *p < 0.05 vs. sham group. ^#^*p* < 0.05 vs. NVHL group.

Neonatal ventral hippocampal lesioned rats showed a decreased average PPI compared to sham control groups, while this change was reversed by microinjection of NRG-1 (1000 ng/0.5 μl) into the PFC of NVHL rats [Two-way ANOVA: group: *F*_(1,48)_ = 53.55, *p* < 0.01, treatment: *F*_(2,48)_ = 2.81, *p* < 0.05, group × treatment: *F*_(2,48)_ = 2.74, *p* < 0.05; *Post-hoc* test (average PPI): 500 ng/0.5 μL: *p* = 0.081; 1000 ng/0.5 μL: *p* < 0.05, Figure 4B]. Regarding startle amplitude, it was unchanged among all groups [Two-way ANOVA: group: group: *F*_(1,48)_ = 0.49, *p* = 0.49, treatment: *F*_(2,48)_ = 0.57, *p* = 0.57, group × treatment: *F*_(2,48)_ = 0.039, *p* = 0.96, Figure 4C].

## Discussion

In the present study, we have demonstrated that post-pubertal NVHL rats exhibit a decrease in the levels of both NRG-1 and p-erbB4 in the PFC. Moreover, we have observed that intra-cortical microinjection of NRG-1 was able to attenuate the PPI deficits in post-pubertal NVHL rats.

NRG-1 expression in the central nervous system has been detected in many regions including the PFC, hippocampus, cerebellum, and substantia nigra, in both humans and rodents (Chaudhury et al., 2003; Law et al., 2004; Bertram et al., 2007). ErbB4 is the only receptor with a high affinity for NRG-1 (Mei and Xiong, 2008), and is mostly located in the GABAergic neurons in the PFC (Bean et al., 2014). Postmortem study has showed that cortical NRG-1 level is decreased in schizophrenia patients (Bertram et al., 2007; Marballi et al., 2012). NRG-1 or erbB4 knock out mice exhibit schizophrenia-like behaviors such as disruption of PPI, latent inhibition and cognitive deficits (Stefansson et al., 2002; Falls, 2003; Hashimoto et al., 2004; Rimer et al., 2005; O’Tuathaigh et al., 2007). From these findings, dysfunction of NRG-1/erbB4 signaling may be associated with development of schizophrenia. In the present study, NVHL rats, which also exhibit schizophrenia-like behaviors (Sams-Dodd et al., 1997; Becker et al., 1999; Mitazaki et al., 2020), showed significant reduction in NRG-1 and p-erbB4 levels in the PFC (Figures 2, 3), suggesting that NRG-1/erbB4 pathway in the PFC of NVHL rats was impaired. Previous study showed that nrg-1 gene in the mPFC was unchanged between sham and NVHL rats (Swerdlow et al., 2013). These paradoxical results may be attributed to different methods of NRG-1 quantification. The brain mapping analyzer is equipped with a photomultiplier tube as a detector and outperforms the quantitative linearity of the image analyzer used in high-sensitivity TV cameras by more than two orders of magnitude and the sensitivity of high-performance liquid chromatography with electrochemical detector by more than three orders of magnitude (Sutoo et al., 1988). Glutamatergic neurotransmission regulates shedding of the ectodomain of the NRG-1 precursor, and releases the activated NRG-1 through activation of glutamate receptors/protein kinase C (PKC)/a disintegrin and metalloproteinase families (Iwakura et al., 2017). Our previous studies showed that NVHL rats show decreased phosphorylation of glutamate receptors and PKC in the PFC (Yabuki et al., 2013, 2019). Thus, we think that the reduction of NRG-1 in the PFC of NVHL rats may be associated with dysfunction of glutamatergic system in the PFC. Previous studies have suggested that NRG-1 levels in the PFC are significantly correlated with a number of genes, including csnk1e (casein kinase 1 epsilon), grid2 (glutamate receptor delta-2), comt (catechol-*O*-methyltransferase) in the PFC and NGR-1 levels in other brain regions such as the nucleus accumbens and ventral hippocampus (Swerdlow et al., 2012). Therefore, it is possible that reduced NRG-1 in the PFC does not directly contribute to NVHL-induced PPI impairment, but rather may result by affecting the expression of these genes or by affecting the expression of NRG-1 in other brain regions. This possibility is the subject of future research. Administration of NRG-1 has been found to rapidly promote GABA release in the cortex, and modulates functions of glutamatergic neurons (Woo et al., 2007; Vullhorst et al., 2015). Activation of GABAergic system in the PFC has been reported to improve PPI deficits in animal models for schizophrenia (Bortolato et al., 2007; Fejgin et al., 2009). Disruption of PPI is a translational endophenotype of schizophrenia modeling the pre-attentional and sensori-motor impairments (Lipska et al., 1995). A previous study reported that NVHL rats have a reduction in GAD67 gene expression, a GABA neuron marker, in the PFC (Lipska et al., 2003). Thus, we hypothesized that intra-cortical microinjection of NRG-1 may improve NVHL-induced PPI deficits. Our study showed that the microinjection of NRG-1 (1000 ng/0.5 μl) into the PFC (Figure 4A) partially reversed NVHL-induced PPI deficits (Figure 4B). These present results and previous studies suggest that decreased the levels of NRG-1 and p-erbB4 in the PFC may be involved in PPI deficits in post-pubertal NVHL rats, and this mechanism may be associated with GABAergic system. However, we did not assess the changes in GABA release in the PFC of NVHL rats after NRG-1 administration which we will examine in a future study. Moreover, previous study has reported a correlation between lesion size and PPI deficits (Swerdlow et al., 2013). However, we did not perform the quantification of lesion size and assessment of the relationship between lesion size and NRG-1/erbB4 effects, and these questions also need to be addressed in future studies.

Animal models of psychiatric disorders have many limitations and cannot fully reflect the conditions observed in humans (Białoń and Wa̧sik, 2022). The NVHL model is inadequate for assessing the functionality of protein changes observed in schizophrenia patients, and future studies are needed to determine whether similar results can be obtained in transgenic animals.

In summary, this study revealed that alteration of NRG-1/p-erbB4 in PFC may play a role in abnormal behaviors observed in post-pubertal NVHL rats. Based on our data in this putative animal model for schizophrenia-like phenotype, we speculate that at least some of the sensorimotor gating deficits in schizophrenia patients may be due to reduction in NRG-1 in the PFC.

## Data availability statement

The original contributions presented in this study are included in the article/supplementary material, further inquiries can be directed to the corresponding author.

## Ethics statement

This animal study was reviewed and approved by Ethics Committee of Animal Experiment in Tohoku Medical and Pharmaceutical University.

## Author contributions

KW, ON, KT, and LS: writing—review and editing. KW, ON, and KT: conceptualization. S-iK, SM, HO, K-iW, KT-N, and MI: methodology. KW, SM, and HO: formal analysis. KW and KT: visualization. ON: resource. LS, RQ, and TT: supervision. All authors interpreted the results and read and approved the final version of the manuscript.
